# Comprehensive Care for Wildfire Survivors in Los Angeles, 2025

**DOI:** 10.1002/puh2.70146

**Published:** 2025-10-28

**Authors:** Kojima N.

**Affiliations:** ^1^ Department of Population and Public Health Sciences Keck School of Medicine University of Southern California Los Angeles California USA

## Abstract

From January 7 to 31, 2025, Los Angeles experienced a series of destructive wildfires. This is a representative narrative short report of patients who were affected by the wildfires and the services they requested. This short report was written to provide narratives of patients who were affected by the natural disaster, details of hardships faced in its aftermath, a summary of services that were available through local disaster centers, and insights for future natural disaster preparedness planning efforts.

On the morning of January 7, 2025, I saw smoke blowing from the Pacific Palisades toward the ocean. By late morning, flames and columns of smoke were visible for miles. Due to dry weather conditions and hurricane‐force winds reaching speeds of up to 100 miles per hour, the fire rapidly spread [1]. It eventually burned over 23,000 acres, destroying over 6500 structures and quickly displacing many people.

During the fires, I cared for patients at an immediate care clinic and a local disaster relief center. Here, I describe the general challenges patients faced during this period to provide narratives of survivors of a major metropolitan natural disaster, list resources that were helpful for displaced people that would be useful in other disasters for public health preparedness, and add my personal insights into immediate care delivery.

At an immediate care clinic and a local disaster relief center, all of the patients I evaluated were either directly or indirectly affected by the fires: Some had burns and smoke inhalation injuries and others had physical trauma due to falls or automobile accidents caused during rapid evacuations. Patients had acute on chronic disease exacerbations due to diseases like asthma from smoke and toxin exposure, many had lost necessary medicines, and all had acute stress.

During this period, I saw hundreds of patients. Here, I provide the following patient narratives as they highlight both the resiliency and profound challenges caused by the wildfires. Many patients were directly affected by the fires. One patient I cared for at the disaster relief center lost her home and her arthritis medications in the fires. She was working at another diaster relief station and came to ask for medication refills during her break. After she told us what medications she needed and confirmed that her medications were refilled, she returned to her station with a non‐profit organization assisting others displaced by the fires. Many patients requested assistance replacing dentures, glasses, orthotics, breathing machines, and other durable medical equipment.

Other patients were affected by one of the sequelae of the fires: crime. As most evacuees had to store their possessions in their cars, theft was prevalent. A patient who recently had a heart attack had his essential medications stolen from his car along with all his other possessions. This happened to many of the patients I saw who had conscientiously evacuated with their medications and medical equipment, only to have their vehicles broken into after escaping the fire. One patient lamented that she had just fled the war in Ukraine only to have to escape from “another war zone.”

Despite obtaining prescriptions, there were barriers. Many patients had challenges accessing medications due to their insurance companies denying their refills as “too early.” Despite communications with insurance providers who said they would make exceptions for those affected by the wildfires [2], claims were still denied. Some patients paid out‐of‐pocket costs; others went without medications they decided were “not essential,” such as blood pressure medications, due to cost. Calls with pharmacies sometimes helped if the pharmacists would approve emergency overrides; however, medical providers often needed to advocate for this to happen. The offices of medical providers were also lost in the fires so it was not always possible to identify and notify their providers.

The immediate downstream effects of the widespread evacuation were already present. Where hygiene and personal space were more limited, like in densely populated shelters, diseases spread. There was a norovirus outbreak at one wildfire shelter and influenza and other respiratory viruses circulating in others that I directly managed [3]. Despite collaboration with the Public Health Department, onsite coordination was difficult because medical notes were kept on paper charts and the volunteer staff had regular turnover, which made consistent communication challenging.

To prepare for the next major metropolitan natural disaster, it is essential that local preparedness protocols and drills are regularly conducted with multidisciplinary teams, stores of essential medications and durable medical equipment that can be quickly distributed are shored up, protocols are present for increased law enforcement presence to create safe areas for evacuees, predetermined overrides for medication fills in areas with natural disasters are in place to ensure that there are reduced gaps in medication coverage, and emergency shelter sites have standard operating procedures in place to accommodate a large number of displaced people. This report is limited to the patients and personnel that I directly interacted with in my work.

Partnerships between the cities, county, state, and federal government facilitated access to services for people displaced by the wildfires in an expedient manner (Table 1). Public–private partnerships gave rise to mass donations of time, material goods, and physical space [4]. In addition to providing access to medicines, personal protective equipment, hand sanitizer, and information, humanizing survivors by treating them with dignity played a therapeutic role. For some, it helped to be able to share their traumatic experiences with someone who would listen.

**TABLE 1:** A non‐complete list of useful services for wildfire survivors.

**Acute**
Clothing
Communication
First aid and triage
Food and water
Medical and mental health
Medicines
Personal hygiene
Pet resources
Reunification
Shelter
**Semi‐acute**
Dentures
Glasses
Longer term housing without price gouging
Orthotics
Other durable medical equipment
Personal protective equipment
Transportation and access
**Other services** [4, 5, 6]
Animal care
Assessment of property value
Building permits
Car identification
Child support
Debris removal
Document and identification replacement (birth/death certificates, vital records, marriages licenses, social security cards, driver's licenses or other identification cards, passports, home documents, supplemental nutrition assistance program documents, Medicare Cards, Green cards, Military Documents, ATM & Credit Cards)
Emergency loans
Employment transition
Environmental services
Homeless initiatives
Immigration services
Income support
Insurance claims
Loans to repair or replace property
Mail forwarding
Medical Examiner
Pre‐employment and employment support
Returning to property
School
Tax reassessment (income tax documents) and relief
Water safety

And we should listen. The worsening environmental disasters are a global problem. There are not only wildfires but also increasing numbers of hurricanes, floods, and other natural and unnatural catastrophes that all greatly impact human health. It is necessary to bolster emergency preparedness, not only for ourselves and our loved ones, but also at the community level and higher, to ensure we not only survive but are also able to mitigate harm, rebuild, and hopefully prevent diasters in a sustainable way.

## Author Contributions

 NK drafted and edited this manuscript.

## Disclosure

NK has no disclosures to report. An IRB review was not requested, as this was not research.

## Conflicts of Interest

The author declares no conflicts of interest.

## Data Availability

The data that support the findings of this study are available from the corresponding author upon reasonable request.
